# Age-Related Reduced Somatosensory Gating Is Associated with Altered Alpha Frequency Desynchronization

**DOI:** 10.1155/2015/302878

**Published:** 2015-08-31

**Authors:** Chia-Hsiung Cheng, Pei-Ying S. Chan, Sylvain Baillet, Yung-Yang Lin

**Affiliations:** ^1^Department of Occupational Therapy and Graduate Institute of Behavioral Sciences, Chang Gung University, Taoyuan, Taiwan; ^2^Healthy Aging Research Center, Chang Gung University, Taoyuan, Taiwan; ^3^McConnell Brain Imaging Center, Montreal Neurological Institute, McGill University, No. 3801, University Street, Montreal, QC, Canada H3A 2B4; ^4^Institute of Brain Science, National Yang-Ming University, Taipei, Taiwan; ^5^Department of Neurology, National Yang-Ming University, Taipei, Taiwan; ^6^Institute of Physiology, National Yang-Ming University, Taipei, Taiwan; ^7^Institute of Clinical Medicine, National Yang-Ming University, Taipei, Taiwan; ^8^Laboratory of Neurophysiology, Taipei Veterans General Hospital, Taipei, Taiwan; ^9^Department of Neurology, Taipei Veterans General Hospital, Taipei, Taiwan

## Abstract

Sensory gating (SG), referring to an attenuated neural response to the second identical stimulus, is considered as preattentive processing in the central nervous system to filter redundant sensory inputs. Insufficient somatosensory SG has been found in the aged adults, particularly in the secondary somatosensory cortex (SII). However, it remains unclear which variables leading to the age-related somatosensory SG decline. There has been evidence showing a relationship between brain oscillations and cortical evoked excitability. Thus, this study used whole-head magnetoencephalography to record responses to paired-pulse electrical stimulation to the left median nerve in healthy young and elderly participants to test whether insufficient stimulus 1- (S1-) induced event-related desynchronization (ERD) contributes to a less-suppressed stimulus 2- (S2-) evoked response. Our analysis revealed that the minimum norm estimates showed age-related reduction of SG in the bilateral SII regions. Spectral power analysis showed that the elderly demonstrated significantly reduced alpha ERD in the contralateral SII (SIIc). Moreover, it was striking to note that lower S1-induced alpha ERD was associated with higher S2-evoked amplitudes in the SIIc among the aged adults. Conclusively, our findings suggest that age-related decline of somatosensory SG is partially attributed to the altered S1-induced oscillatory activity.

## 1. Introduction

Despite the continuous attention to the age-related changes in the higher hierarchical cognitive function, recent imaging studies have shown that the early-phase perceptual process, for example, cortical inhibition or sensory gating (SG), is also modulated by aging [1–7]. Most importantly, this cortical disinhibition has been linked to the aberrant neuropsychological or behavioral performance [5, 8, 9].

Compelling evidence shows that electrical stimulation to the median nerve elicits synchronous cortical reactivity in the primary somatosensory cortex (SI) and bilateral secondary somatosensory cortex (SII) [10–14]. By using paired-pulse electrical stimulation, in which two stimulus pulses in close succession are presented, it has been extensively applied to study the functional integrity of cortical inhibition or excitability in various clinical disorders, such as schizophrenia [15], traumatic brain injury [16], complex regional pain syndrome [17], dystonia [18], migraine [19], and aging [3, 5]. Quantitatively, SG is measured as the amplitude ratio of stimulus 2-evoked responses over stimulus 1-evoked responses (S2/S1) [20]. A larger S2/S1 ratio is indicative of reduced cortical inhibition. With this technique, one previous event-related potential (ERP) study has revealed an age-associated SG defect in the human SI [5]. Very recently, our magnetoencephalographic (MEG) study by applying equivalent current dipole (ECD) modeling has demonstrated that the neurophysiological responses of the SII are particularly vulnerable to aging in terms of cortical SG [2].

In addition to phase-locked evoked responses, non-phase-locked brain oscillations might be implicated in the basic somatosensory perceptual processing [21]. Cortical oscillation is considered to reflect the excitability of thalamocortical systems and can be modulated by exogenous or endogenous events [22]. Event-related desynchronization (ERD) represents a decrease in synchronization of a specific frequency in relation to the activation of the somatosensory system [23–25]. Previous studies have reported significant alpha and/or beta ERD in the SI [26–29] and SII [30–32] following electrical or laser stimulation among the young healthy adults. However, it still remains unclear whether the somatosensory ERD is affected by physiological aging.

Although previous literature has demonstrated reduced somatosensory SG as a function of age, the possible contributing factors or variables are obscure. An association between oscillatory activity and cortical excitability in the sensorimotor cortex has been shown [31, 33, 34]. Here, we were intrigued to examine whether S1-induced ERD activity serves as a possible factor to account for the age-related alterations in the S2-evoked excitability.

Specifically, this MEG study aimed (1) to investigate the effects of aging on somatosensory cortical alpha and beta ERD induced by median nerve stimulation and (2) to determine the relation between S1-induced ERD and S2-evoked responses. Our working hypothesis was that the elderly might demonstrate reduced somatosensory SG and ERD magnitude. Finally, we predicted that a less-suppressed S2-evoked activity in the aged adults might be associated with deficient S1-induced ERD.

## 2. Methods

### 2.1. Participants

Eighteen young (mean 23.7 ± 0.9 years) and fifteen elderly (mean 68.5 ± 2.2 years) healthy male volunteers participated in this study. All subjects were right-handed with no history of neurological or psychiatric disorders. The majority of these participants were from our previous research project [2]. The Institutional Review Board of the Taipei Veterans General Hospital approved the protocol, and informed consent was obtained from all subjects.

### 2.2. Paradigm

The left median nerve was stimulated at the wrist with 0.2 ms constant-current square-wave pulses by an electrical stimulator (Konstantstrom Stimulator, Schwind, Erlangen, Germany). Stimulus intensity was set at 20% above the motor threshold for eliciting a visible twitch of the abductor pollicis brevis muscle (young = 4.4 ± 0.1 mA, elderly = 4.6 ± 0.1 mA; *P* = 0.29; unpaired two-tailed* t*-test). Stimuli were delivered in pairs with an interstimulus interval (ISI) of 0.5 s and an interpair interval of 8 s. The ISI of ~0.5 s allowed us to simultaneously examine the whole somatosensory system, including SI and bilateral SII areas [2, 15, 31]. Subjects were asked to ignore the stimulation and focus on a silent video, in which way we could examine the preattentive responses without contamination by anticipation effects.

### 2.3. MEG Recordings

The cortical magnetic fields were recorded with whole-head 306-channel MEG (Vectorview, Elekta Neuromag, Helsinki, Finland). The data from planar gradiometers of this device, which detect the largest signal directly above the activated cerebral areas [35], were analyzed. The coil locations in relation to the anatomical landmarks (left preauricular point, right preauricular point, and nasion) were determined with a 3D digitizer.

The MEG signals were digitized at a sampling rate of 500 Hz, with an online bandpass of [0.1, 200] Hz. An interval of 0.5 s, including a prestimulus baseline of 0.1 s, was evaluated. Epochs contaminated by prominent electrooculogram signals (>300 *μ*V) and MEG artifacts (>3000 fT/cm) were automatically excluded from averaging. At least 100 artifact-free evoked S1 and S2 responses were averaged online.

### 2.4. Source Estimation

The averaged data were offline filtered with a bandpass of [0.1, 120] Hz and a 100 ms baseline correction. We applied a distributed minimum norm estimate (MNE) source modeling to reconstruct evoked responses and identified three regions of interest (ROIs): SI and contralateral (SIIc) and ipsilateral (SIIi) secondary somatosensory cortex.

The modeling of the cortical spatiotemporal dynamics of somatosensory evoked responses was obtained with Brainstorm [36]. The segmentation of head tissues from individual T1-weighted magnetic resonance imaging (MRI, GE Discovery MR 750 3T with TR 9.4 ms, TE 4 ms, recording matrix 256_256 pixels, field of view 256 mm, slice thickness 1 mm) volume data was obtained with BrainVisa (http://brainvisa.info/). The representation of folded cortical surface was used to resolve the forward problem by applying an overlapping-sphere model, which derives the strength of a set of electric dipoles located at the cortical surface [37]. For each participant, cortically constrained source imaging was performed using the depth-weighted MNE [38, 39] model of Brainstorm, with default parameter settings, over a set of ~7500 elementary current dipoles distributed over the individual cortical envelope. The individual source maps were geometrically registered to the Montreal Neurological Institute (MNI) brain template (Colin27) using Brainstorm's multilinear registration technique, with default parameters.

The MNE source maps were obtained for each participant and each stimulus condition and group-averaged onto the aligned cortical surface of the Colin27 brain template. Based on the grand-averaged waveform time series and cortical activation, a cluster of 30 vertices corresponding to 4-5 cm^2^ was manually selected to define each ROI.

The time-resolved magnitude of each elementary source was normalized to its fluctuations over baseline, yielding a set of *Z*-scored time series at each cortical location [40, 41]. The *Z*-score values were rectified to detect absolute magnitude changes above baseline levels, and peak responses to S1 and S2 of each ROI were extracted for subsequent analysis. The degree of SG was quantified as the ratio of the strength of S2 divided by S1.

### 2.5. Spectral Analysis

Each raw single trial in the selected ROIs was analyzed by using Morlet wavelet-based time-frequency approach in Brainstorm software. Epochs of 2.5 s duration with 1.0 s preceding S1 and 1.0 s following S2 were created. Due to the longer peak latency of ERS (~ ≧0.7 s) [23, 25] and our design of 0.5 s ISI, this study specifically focused on the S1-induced ERD responses.

In the ERD computation, the alpha (8 to 13 Hz) and beta (14 to 30 Hz) bands which exhibited the most reduced activity (0.0 s to 0.5 s following S1) were identified. The averaged baseline power density (−0.9 s to −0.5 s before S1) was calculated after the *Z*-score correction. We selected peak amplitudes of the most reactive frequency bands (2 Hz) [25] of alpha and beta rhythms and compared them with respect to the baseline power level in each individual.

### 2.6. Statistical Analysis

All the data were presented as mean ± standard error of the mean (SEM). Prior to the statistical analysis, all variables were normal distributed as indicated by the Kolmogorov-Smirnov test (*P* > 0.05). The effects of age on SG and ERD were calculated by independent *t*-test. The relationship between S1-induced ERD and S2-evoked responses was evaluated by Pearson's correlation coefficients. All the analyses were performed with the SPSS statistical package (version 13.0). *P* values of <0.05 were set as the significant threshold.

## 3. Results

### 3.1. Somatosensory SG

**Figure 1:**
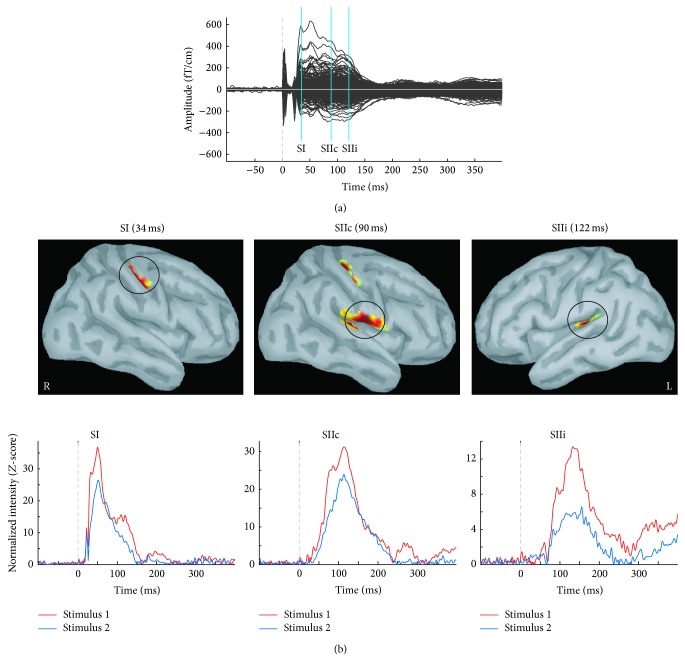
(a) Somatosensory evoked fields to electrical stimulus 1 in one young representative subject. The early primary somatosensory response (SI) is followed by longer latency contralateral (SIIc) and ipsilateral (SIIi) secondary somatosensory responses. (b) Upper panel: three regions of interest (ROIs, 4 to 5 cm^2^) on the Montreal Neurological Institute Colin27 brain template; lower panel: the temporal dynamics of the minimum norm estimate (MNE) in response to stimulus 1 and stimulus 2 are extracted from the selected ROIs.

Figure 1(a) shows the butterfly plot of somatosensory evoked responses to S1 in one young participant. The prominent P35m of SI was followed by the longer latency responses in the SII regions. The upper panel of Figure 1(b) exhibits the MNE source reconstruction of the selected latencies of 34 ms, 90 ms, and 122 ms. The SI responses were generated in the postcentral wall of central fissure and the SII responses were generated in the upper bank of the Sylvian fissure in the parietal operculum. The lower panel of Figure 1(b) demonstrates the source strength as a function of time in these three ROIs of the same subject. The S2-evoked responses (blue trace) were smaller than S1-evoked responses (red trace).

**Figure 2:**
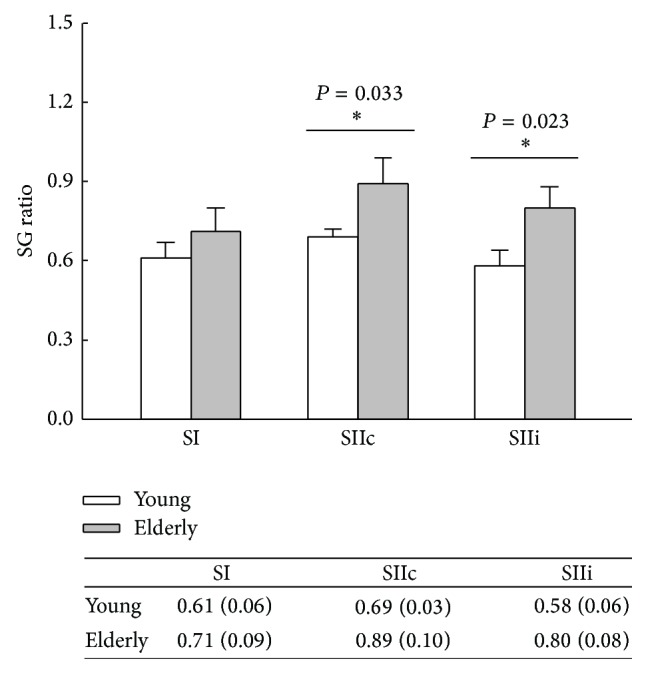
The statistical comparisons between young and elderly individuals in terms of sensory gating (SG) ratio in SI, SIIc, and SIIi areas. The bar above each column indicates the standard error of the mean (SEM).

SG ratios were calculated from each individual and compared between groups. The statistical results show significant higher SG ratios of SIIc (*P* = 0.033) and SIIi (*P* = 0.023) in the elderly group compared to the younger group (Figure 2).

### 3.2. Somatosensory ERD

**Figure 3:**
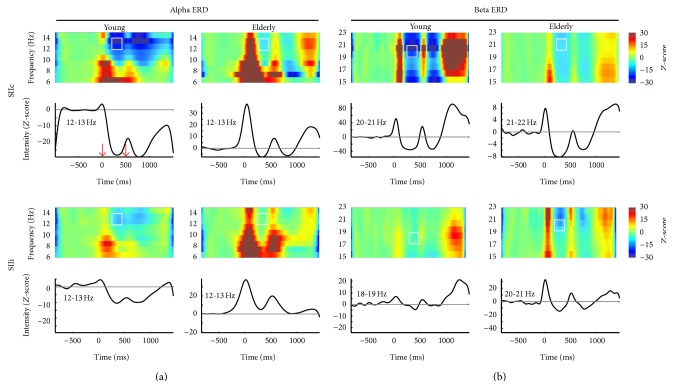
Time-frequency power analysis of the SIIc and SIIi for the alpha (a) and beta (b) frequency bands. The spectrograms between 6 and 14 Hz within alpha range and between 15 and 23 Hz within beta range are displayed. The plots below each time-frequency map exhibit the grand-averaged time course of event-related desynchronization (ERD) reactivity in the most reactive frequency bands (2 Hz) with respect to baseline power. The red arrows correspond to the onset of electrical stimulation. The peak values of induced ERD following stimulus 1 (white squares) are extracted for the subsequent analysis.

Due to the obvious between-group differences in the bilateral SII regions, we then concentrated on the effects of aging on S1-induced ERD reactivity in these neural substrates.  Figure 3 displays the grand-averaged time-frequency representations of alpha (Figure 3(a)) and beta (Figure 3(b)) rhythms over the time interval of 1.0 s before S1 and 1.0 s after S2 in SIIc and SIIi. The plots below each spectral representation exhibit the grand-averaged temporal dynamics of the most reactive frequency ranges (2 Hz) in terms of alpha and beta oscillations. The white squares show the most prominent S1-induced ERD reactivity.

Table 1 lists the averaged ERD-reactive frequency for alpha and beta rhythms. There were no significant differences between young and elderly participants regarding the ERD-reactive frequencies.

**Figure 4:**
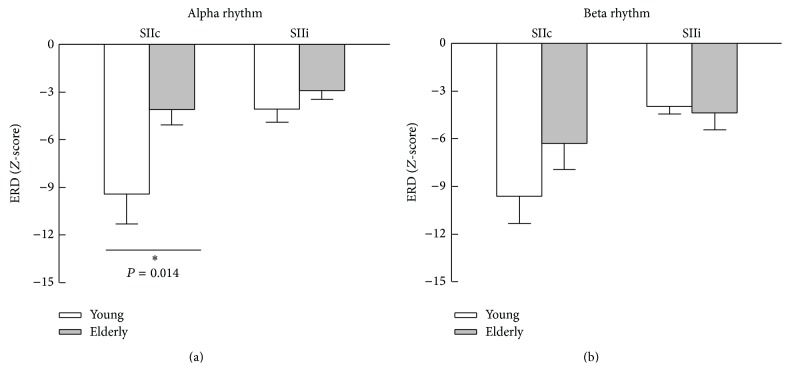
Mean values of stimulus 1-induced ERD peaks for alpha and beta rhythms. The bar above each column indicates the SEM.

Figure 4 shows the mean peak values of alpha and beta ERD with respect to the baseline power. Compared to the younger subjects, the elderly exhibited significantly reduced amplitude of alpha ERD in SIIc (*P* = 0.014). We did not find significant between-group differences in terms of alpha ERD in SIIi and beta ERD in bilateral SII regions.

### 3.3. Correlation between S1-Induced ERD and S2-Evoked Responses

**Figure 5:**
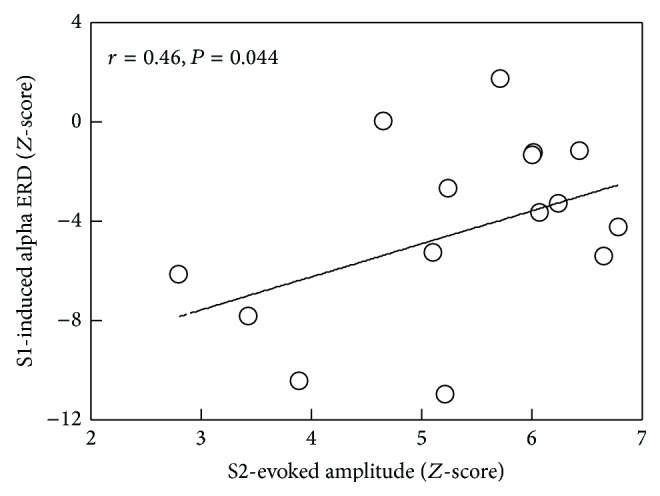
Association between stimulus 1- (S1-) induced alpha ERD and stimulus 2- (S2-) evoked response in the SIIc among the elderly participants.

Given the pronounced reduction of alpha ERD in the elderly adults, we then tested whether S1-induced oscillatory responses influence the performance of S2-evoked reactivity. Lower S1-induced alpha ERD was associated with higher S2-evoked amplitude in SIIc (*r* = 0.46, *P* = 0.044) among the elderly participants, as shown in Figure 5.

## 4. Discussion

To obtain insight into the age-related alterations of cortical inhibition in the human somatosensory system, we applied paired-pulse electrical stimulation to the left median nerve, and our results revealed several important findings. Firstly, by using MNE source modeling, the elderly demonstrated reduced SG in bilateral SII regions, replicating our previous ECD results. Secondly, based on the time-frequency approach, we found age-related reduction of alpha ERD amplitude in the SIIc. Lastly, higher S2-evoked responses were associated with reduced S1-induced alpha ERD amplitudes among the elderly participants, especially in the SIIc area. A higher S2-evoked response could be regarded as poor suppression to repetitive stimuli.

The present MNE data demonstrated higher SG ratios of bilateral SII areas in the elderly adults compared to the younger participants, which is consistent with our previous results [2]. These findings highlight somatosensory SG as a prominent manifestation during the late-age stage, particularly in the higher-order SII neural substrates. By calculating the number of dendritic spines and synaptic density, it has been reported that the association areas are more vulnerable to aging processing compared to the primary sensory cortices [42]. Moreover, one functional magnetic resonance imaging study with dynamic causal modeling has delineated that somatosensory information conveyed in hierarchy but in parallel from thalamus to both SI and SII [43]. This observation supported our notion that SII might be independently affected by aging processes.

By using short stimulus onset asynchrony (i.e., 30 ms), one previous ERP report has shown age-associated decline of SG in the SI. The reduction of cortical inhibition also correlated with impairment of two-point discrimination performance in the aged participants [5]. Although the early somatosensory evoked response, N20m, has been proven to recover to the saturated amplitude with an ISI of less than 100 ms [44, 45], our selection of ISI of 500 ms allowed us to examine P35m of SI and SII simultaneously [15]. Most importantly, a recent MEG study has revealed a superior signal-to-noise ratio for P35m at an ISI of 500 ms than other tested conditions, which lent support to the rationale of our design [29]. Collectively, our results of age-related somatosensory cortical disinhibition were in favor of* inhibition deficit* hypothesis in aging brains [6, 7, 46–49].

To the best of our knowledge, this is the first MEG study to investigate the effects of aging on spectral power changes in somatosensory alpha and beta oscillations. Our results demonstrated that the elderly showed reduced alpha ERD power in the SIIc. This significant finding particularly specific to alpha component indicated that this frequency band is strongly related to the function of the somatosensory cortex and has an apparent influence on information processing of the human brain [50]. The spontaneous alpha oscillatory activity could be replaced by a desynchronized activity following exogenous stimuli, such as median nerve stimulation [51]. In the present research, the desynchronized alpha rhythms might shift their role from idling activity to the processing of sensory inputs, likely through the mechanisms of changes in the local neural interactions [25, 52]. From this standpoint, we speculated that attenuation of alpha ERD in the aged adults was likely due to the age-associated decline of somatosensory information processing. One might argue that alpha ERD could be modulated by stimulus intensity [28]. However, due to the similar electric stimulus intensity provided to the young and elderly groups (4.4 ± 0.1 mA versus 4.6 ± 0.1 mA), the age-related cortical power differences are unlikely to be a consequence of differential stimulus condition.

The underlying mechanisms regarding the association between event-induced neural oscillations and event-evoked responses remain unclear. Previous studies have independently investigated stimulus-induced ERD or stimulus-evoked activity by using paired-pulse somatosensory stimulation [17, 29, 53, 54]. Our present research attempted to relate S1-induced suppression of alpha rhythm to S2-evoked cortical excitability. It is conceptualized that more alpha suppression refers to better functioning of information processes; on the other hand, an increased response to the second stimulation within paired-pulse paradigms indicates an insufficient gating ability. We found that more S1-induced desynchronization of alpha oscillation, especially in SIIc, was associated with less S2-evoked amplitude of evoked response in the older adults. This observation suggests that oscillatory activities could, to some extent, account for the age-related decline of somatosensory SG.

Up to the present, it is extremely unclear why the association between S1-induced alpha ERD and S2-evoked amplitude was observed only in the SIIc region. One possible account is that, compared to the SIIc, SIIi signals usually showed poorer signal-to-noise ratios. Another interpretation is from the neuroanatomical and functional neuroimaging evidence. It has been suggested that SIIc and SIIi receive parallel projections from thalamus [43], which accounts for the reason that bilateral SII regions were venerable to aging, whereas the SI was relatively preserved. However, the mechanisms of ERD generation are more complicated by which reciprocal interactions between thalamus and somatosensory cortices are involved. In the present study, the observed association between S1-indiced ERD and S2-evoked amplitude was restricted in the SII area. This finding did not imply that unilateral decreased S1-induced ERD modulated bilateral S2-evoked excitability. Here, we proposed a relation between age-related somatosensory induced and evoked responses, particularly in the SIIc region. It merits further investigation to determine other mechanisms underlying the age-related reduction of somatosensory SG.

Various neurophysiological studies have supported the argument that GABAergic inhibitory dysfunction is involved in physiological aging [55, 56]. For example, it has been shown that the age-related GABAergic degradation in the hippocampus was due to a selective loss of GABAergic interneurons [57, 58]. In humans, by intravenous injection of scopolamine, a cholinergic antagonist, the participants exhibited increased amplitudes of P50m during repetitive auditory inputs [59]. Moreover, the inhibition of somatosensory activation, particularly P35m in SI and bilateral SII regions, was modulated by GABAergic agonist lorazepam [60], suggesting the GABAergic regulation is related to inhibitory processing. Although our investigation at a system level was unable to verify the molecular mechanisms in terms of age-related defects of alpha ERD and SG, the current data extended the previous findings to highlight GABAergic alternations in human somatosensory information processing.

## 5. Conclusions

By using MEG and paired-pulse electrical stimulation to examine the time-frequency characteristics of somatosensory cortical processing, our results revealed age-related decline of SG and alpha ERD. Notably, an association between neural oscillations and evoked cortical excitability was found in the SIIc region, which indicated that lower S1-induced alpha ERD may be related to higher S2-evoked amplitude (insufficient gating). Taken together, these results suggest that the age-related decrease of somatosensory SG is related to the altered oscillatory activity. This paired-pulse protocol may also serve as an objective measure to assess the effects of training or intervention on somatosensory functioning in terms of cortical neural filtering ability in rehabilitation settings.

## Figures and Tables

**Table 1:** Mean (SEM) ERD reactive frequency for alpha and beta rhythms.

Region	Alpha		Beta	
	Young	Elderly	Young	Elderly
SIIc	11.3 0.24	11.4 0.24	19.0 0.82	19.1 0.87
SIIi	10.9 0.83	11.0 0.35	17.7 0.69	17.9 0.63
